# Facile Determination of the Poisson’s Ratio and Young’s Modulus of Polyacrylamide Gels and Polydimethylsiloxane

**DOI:** 10.1021/acsapm.3c03154

**Published:** 2024-02-05

**Authors:** Ariell Marie Smith, Dominique Gabriele Inocencio, Brandon Michael Pardi, Arvind Gopinath, Roberto Carlos Andresen Eguiluz

**Affiliations:** † Department of Materials Science and Engineering, School of Engineering, University of California, Merced, 5200 North Lake Road, Merced, California 95344, United States; ‡ Department of Bioengineering, School of Engineering, University of California, Merced, 5200 North Lake Road, Merced, California 95344, United States; § Health Sciences Research Institute, University of California Merced, Merced, 5200 North Lake Road, Merced, California 95344, United States

**Keywords:** polyacrylamide hydrogel, polydimethylsiloxane, Poisson’s ratio, Young’s modulus, shear rheology

## Abstract

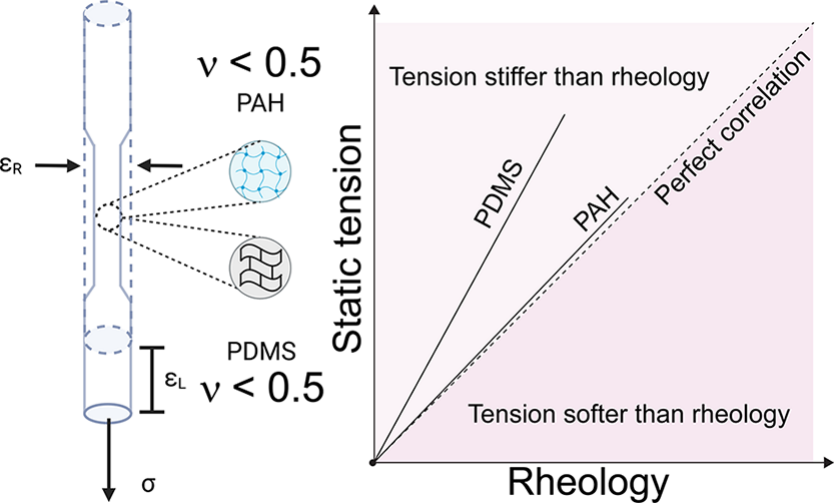

Polyacrylamide hydrogels (PAH gel) and polydimethylsiloxane (PDMS, an elastomer) are two soft materials often used in cell mechanics and mechanobiology, in manufacturing lab-on-a-chip applications, among others. This is partly due to the ability to tune their elasticity with ease in addition to various chemical modifications. For affine polymeric networks, two (of three) elastic constants, Young’s modulus (*E*), the shear modulus (*G*), and Poisson’s ratio (ν), describe the purely elastic response to external forces. However, the literature addressing the experimental determination of ν for PAH (sometimes called PAA gels in the literature) and the PDMS elastomer is surprisingly limited when compared to the literature that reports values of the elastic moduli, *E* and *G*. Here, we present a facile method to obtain the Poisson’s ratio and Young’s modulus for PAH gel and PDMS elastomer based on static tensile tests. The value of ν obtained from the deformation of the sample is compared to the value determined by comparing *E* and *G* via a second independent method that utilizes small amplitude shear rheology. We show that the Poisson’s ratio may vary significantly from the value for incompressible materials (ν = 0.5), often assumed in the literature even for soft compressible hydrogels. Surprisingly, we find a high degree of agreement between elastic constants obtained by shear rheology and macroscopic static tension test data for polyacrylamide hydrogels but not for elastomeric PDMS.

## Introduction

Gels and elastomers have become popular substrates for studies of cell mechanics and mechanobiology, in manufacturing lab-on-a-chip applications, among others.^1−5^ This is in part due to the ease in fabricating these materials into complex shapes, the ability to tune the mechanical properties, and the variety of specifically tailored surface modifications possible. These soft materials may be characterized mechanically at a variety of scales: elastic properties can be determined at small scales using atomic force microscopy (AFM), at larger bulk scales by shear and normal rheology, or by dynamic mechanical analyses using indentation or tension. Since these materials possess strain- and strain-rate-dependent properties and are often hydrated and porous, rheology provides an especially efficient mode of interrogation and analysis.

For small deformations and under the action of weak stresses, gels, and elastomers behave as linear elastic materials. When coarse-grained and probed at the macroscale, these materials may also be approximated as isotropic materials. Linear isotropic elastic materials are characterized by three moduli characterizing different deformation modes – the extensional modulus *K* (addressing extensional properties), the shear modulus *G* (addressing shearing deformations), and the bulk modulus *B* (quantifying response to bulk volumetric compression). These elastic moduli depend linearly on Young’s modulus *E* and are related by Poisson’s ratio ν^5−12^ that quantifies the compressibility of the material and is, therefore, an important material property for soft gels. While *K* and *G* are both measures of the stiffness (or how much the material resists change in shape), ν describes the coupling between axial and transverse deformations. That is, the Poisson’s ratio ν quantifies the degree to which the material contracts laterally (under axial tension) or expands laterally (under axial compression) under applied loads.^13^ Ideal incompressible materials such as rubber, maintain their volume under load (corresponding to high values of *B*) and only change their shape, with ν = 0.5. Soft hydrogels and many elastomers used in bioengineering applications are, however, typically compressible, some very much so. However, the literature addressing experimental determination of ν for important soft materials such as polyacrylamide gels (PAH gel, also sometimes referred to as PAA gels in the literature) and polydimethylsiloxane (PDMS elastomer) is surprisingly limited when compared to the literature reporting values of *E* and *G*.^14−18^ For these materials, the Poisson’s ratio ν deviates significantly from 0.5 and attains values ranging between 0.25 and 0.49,^13−15,19^ depending on the molecular weight and the degree of cross-linking of their constituents.^20^

Attempts to quantify or predict the Poisson’s ratio ν of various polymeric materials have used both experimental approaches as well as theoretical models. For example, the study^14^ by Takigawa et al. used a tensile tester to measure the Poisson’s ratio of 27 wt % PAH gel. The gels were kept hydrated under isothermal conditions using a water bath. Under applied tensile loads, the stretch ratios parallel and perpendicular to the stretched directions were measured, and the value of ν was directly estimated. In the same study, the role of strain rate in impacting Poisson’s ratio was also investigated by varying the strain rate while keeping the PAH gel formulation constant. Interestingly this study reported that ν did not depend on strain rates within investigated conditions and reported a nearly constant value of ν = 0.457. A more recent study by Javanmardi et al.^15^ also using a similar approach, reported that ν values for PAH gels increased with increasing acrylamide concentrations and were far from the usually assumed value of 0.5. Specifically, the authors reported values of ν = 0.24, 0.30, and 0.32 for 3, 4, and 5% acrylamide concentration, respectively. This contrasts, however, with a third study by Boudou et al.^18^ on PAH gel, in which ν was reported to be acrylamide concentration independent, with ν = 0.485, 0.486, and 0.474 for 5, 8, and 10% acrylamide concentration, respectively. These discrepancies in the current literature highlight the importance of systematically characterizing the mechanical response of PAH gels and the need for direct measurement of Poisson’s ratio. This is especially important in applications to cell mechanobiology, where soft hydrogels such as PAH gel are used as substrates. Forces exerted on the material by migrating cells are estimated by measuring deformations and transducing them to stress. For example, techniques based on traction force microscopy (TFM) are often used to study stresses and associated focal adhesion areas in motile cells and parse the data using analytical linear (or nonlinear) constitutive elasticity models.^2,15,21^ Using incorrect values of ν in these models will invariably provide incorrect estimates of stresses exerted by the cells.

Elastic properties of the PDMS elastomer were also measured recently. Laser-engraved grid patterns were used to obtain ν, *E*, and *G* by optically measuring the grid pattern distortion when the specimen was mechanically stretched.^16^ Alternatively, ν can also be determined by exploiting thermal expansion and measuring surface deformations. Using this approach, Müller et al.^17^ estimated ν, and reported values for the silicone elastomers Sylgard 184 and Sylgard 182 of ν = 0.495 and ν = 0.4974, respectively. These values, however, need to be considered carefully, as the time and temperature used for curing, in addition to the formulation (such as the base-to-curing agent ratios), or extraction of nonpolymerized oligomers, are crucial in determining the final structure and mechanical responses of thermally set polymers, such as PDMS elastomers.^22^

The clear need, evident from reviewing the literature, for the characterization of the elastic and viscoelastic properties of substrates used in mechanobiology studies on mammalian cells extends to studies on prokaryotic cells such as bacteria. A recent study found that biofilms of *Serratia marcescens*, *Pseudomonas aeruginosa*, *Proteus mirabilis*, and *Myxococcus xanthus*, have all been found to expand faster on stiffer PAH gel substrates than on softer ones.^2^ TFM measurements showed that the colonies generated transient forces that are correlated over length scales much larger than a single bacterium and that the magnitude of these forces increases with increasing substrate stiffness.^2^ Understanding these trends requires a clear quantification of the (compressional and shear) stress fields in the underlying soft substrates and relating them to intrinsic substrate elastic properties.

Additional motivation comes from theoretical models and simulations that probe how cells sense soft substrates and interact with each other via substrate-mediated elastic communication.^3,23−28^ Cells act as force dipoles, deforming underlying substrates and generating a strain field that can cause nearby cells to reorient and attain energetically favorable configurations. In recent work, we have shown using stochastic agent-based models that Poisson’s ratio ν may play an important role in setting the type and range of these moderate and short-range biophysical interactions.^29,30^ Indeed, more recent work suggests that Poisson’s ratio ν can determine the favorable configurations (both position and orientation) of a pair of dipoles^31,32^ and direct multicellular network formation on elastic substrates. Using experimentally determined values of Poisson’s ratio ν and Young’s moduli *E* rather than approximated values will allow for more realistic theoretical investigations of cell motility, cell–cell interactions, and related mechanobiology problems.

Inspired by the simple macroscopic approach of Pelham and Wang^27^ to estimate Young’s modulus *E* of PAH gel, we describe herein a similarly simple macroscopic method employing tensile tests to measure directly both *E* and ν. We used our methodology to achieve two objectives. First, we directly characterize the Poisson’s ratio and the two Young’s moduli for (three formulations of) PAH gel and (three formulations of) PDMS elastomer, both of which are relevant substrates for mechanobiology and bioengineering applications. This is done using two approaches. The first approach is using a static tension (stretching) test and examining the strain and global deformation from which *E* and ν are directly obtained. The latter is obtained from a change in the sample geometry (dimensions). The shear modulus *G* is then calculated assuming the material to be linearly elastic, isotropic, and undergoing affine deformation. We next use a second independent method based on shear rheology to directly obtain the bulk shear modulus *G* of PAH gel and PDMS elastomer samples using small amplitude oscillatory shear and extrapolate the results to the limit of zero frequency. We find that the Poisson’s ratio may vary significantly from the value for incompressible materials (ν = 0.5), highlighting the need to estimate its value rather than assuming it. Our second objective is to compare the values of the shear moduli from the two independent methods and cross-correlate these values with values reported in the existing literature. Surprisingly, we find a high degree of agreement between shear rheology and macroscopic tension tests for the PAH gel but not for the PDMS elastomer.

Taken together, our study emphasizes the importance of accurately characterizing *E* and ν of gels and elastomers rather than assuming the incompressible value, especially for use in analyzing cell-substrate interactions and cell mechanobiology studies. Our method provides an easy, accessible, and affordable means to achieve this characterization using materials and means commonly found in most laboratories.

## Materials and Methods

### PAH Gel Rod Sample Preparation

An amount of 40% acrylamide (Sigma-Aldrich), 2% bis-acrylamide (Sigma-Aldrich), ammonium persulfate (APS) (Invitrogen), and tetramethylethylenediamine (TEMED) (Thermo-Fisher Scientific), and milli-Q water of the indicated volume shown in Table 1 were mixed together in a 15 mL conical tube, starting with the larger volumes.^6,33,34^ The mixture was then degassed for 10 min. Next, 5 μL of a 10 wt % APS previously prepared and stored at −20 °C was added and the entire solution vortexed. Next, 0.5 μL of TEMED was added and the solution vortexed again. Lastly, the resulting solution was cast, approximately 7 mL, into disposable straws 6 mm in diameter, with one end sealed with parafilm and held under an active vacuum (120 Torr) during the curing time of approximately 30 min. The PAH gel rods were removed from the mold and immersed in excess Milli-Q water (18.2 MΩ cm, TOC < 5 ppm) and allowed to swell overnight at 4 °C, enough to fully swell.^35^

**Table 1:** Formulations Used To Fabricate the PAH Gel; Soft, Intermediate, and Stiff PAH Gel Nomenclatures Are Based on Previously Reported Protocols

nomenclature	acrylamide/bis-acrylamide (%/%)	acrylamide from 40 wt/v% stock (μL)	bis-acrylamide from 2 wt/v% stock (μL)	water (μL)	TEMED (μL)	APS (μL)
soft	5/0.3	125	150	725	0.5	5
intermediate	8/0.2	200	100	700	0.5	5
stiff	8/0.48	200	240	560	0.5	5

### PDMS Elastomer Rod Sample Preparation

PDMS elastomer rods of 50:1, 20:1, and 10:1 (base: curing agent) ratios were created by mixing the appropriate base with curing agent solution using the SYLGARD 184 silicone elastomer kit (see Table 2) and stirred for 5 min or so until fully mixed. The entire solution was degassed for 30 min under vacuum, ensuring that bubbles in the solution were completely removed. 3.5 mL of degassed and premixed solution was pipetted into disposable straws 6 mm in diameter. One straw end was sealed by using a binder clamp. The filled straw was then immediately placed in an oven at 65 °C for 24 h for cross-linking. No ramping temperature rate was used.

**Table 2:** Receipts Used To Fabricate the Elastic PDMS Elastomer

nomenclature	base:curing agent ratios	base (g)	curing agent (g)
50:1	50:1	50	1
20:1	20:1	20	1
10:1	10:1	10	1

The degree of oligomers not incorporated (unreacted) into the cross-linked network of PDMS elastomer samples increases with decreasing base-to-curing ratios (50:1 > 20:1 > 10:1). We measured gel fractions for 50:1, 20:1, and 10:1 base-to-curing agent mass ratios of 84.6 ± 2.5, 96.2 ± 0.27, and 97.7 ± 0.17 wt %, respectively, using chloroform as the extraction solvent. The process is detailed in the SI, and the results are summarized in Figure S1. We do not expect the unreacted oligomers to affect the measured and reported elastic constants, as the relative Young’s moduli value, that is, the ratio of Young’s modulus of the as-cast sample normalized by Young’s modulus of the extracted samples (defined as α = *G*_Rheo_/*G*_Tension_) is α = 1.1 for an extracted mass of 15% or less,^36^ which is within our measured extracted mass fractions.

### Static Tensile Tests

**Figure 1:**
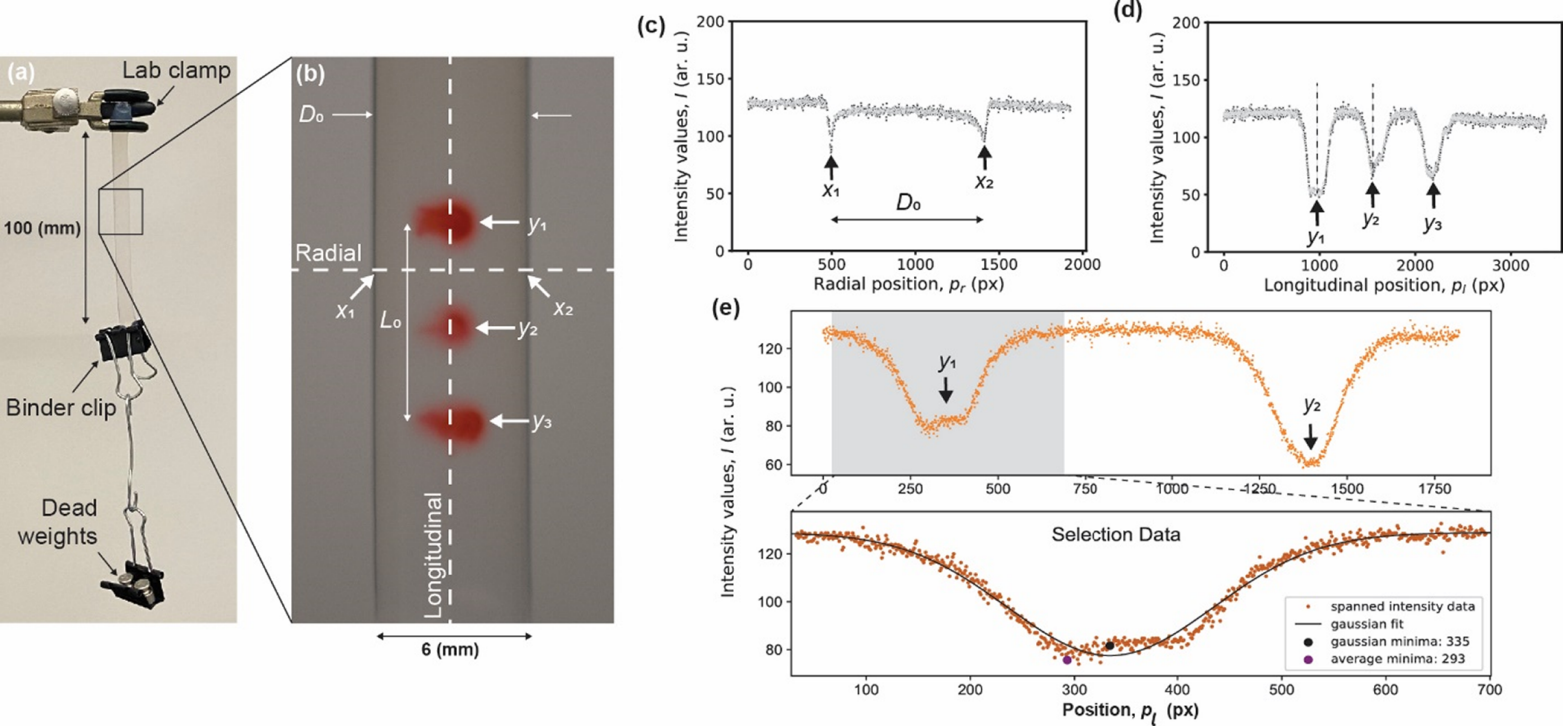
(a) Static tensile test configuration used to extract Poisson’s ratio ν and Young’s modulus *E*. (b) Fiducial markers and line orientations to extract intensity profiles. (c,d) Line intensity profiles used to extract diameter (radial) and length (longitudinal) dimensional changes. (e) Intensity profile analyzer (IPA) region selection to extract the peak position of the intensity profile.

Fully swollen PAH gel rods were first prepared by hydrating them in water. Then, swollen PAH gel and PDMS elastomer rods were carefully mounted on the stretcher device, as shown in Figure 1a, with two clamps attached to each end. The average diameter of our water-swollen PAH gel and PDMS elastomer rods were 6.64 ± 0.65 and 6.54 ± 0.07 mm, respectively. Two to four stains as detailed in the SI were made (inked) using a permanent marker and used as fiducial markers, as seen in Figure 1b. These were made far from the clamps and close to the center of the rods. Inked gels were then subjected to static tensile forces by applying dead weights (see Tables S1 and S2) on the lower end of the PAH gel and PDMS elastomer rods. Using a digital single-lens reflex camera (Nikon, D750) with a macro lens (Nikon, AF-S Micro Nikkor 105) mounted on a tripod, pictures were taken via a wireless intervalometer to prevent mechanical drift. The PAH gel and PDMS elastomer rods were imaged once with each incremental step of dead weights added, ensuring that the fiducial markers were within the field of view. The camera remained static. Images were subsequently postprocessed in FIJI (NIH). Intensity profiles, both horizontal (radial) and vertical (longitudinal), were subsequently traced and analyzed to extract the edge pixel positions via an in-house intensity profile analyzer (IPA) code. Implementation details and use can be found in the SI. From these, we obtained the rod tensile specimen diameter, *D*_*i*_ (in pixels), and the center-to-center distance of the fiducial markers, *L*_*i*_ (in pixels), every time a dead weight (indexed by *i*) was added, Figure 1c,d. The rod tensile specimen diameter *D*_*i*_ and distance *L*_*i*_ were then used to compute the radial and longitudinal engineering strains, ε_R_ and ε_L_ defined in eqs 1a and 1b:
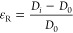
1a
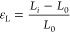
1bwhere *D*_0_ and *L*_0_ are the initial rod tensile specimen diameter and center-to-center fiducial marker length, respectively. The engineering stress σ_*i*_ was also calculated using eq 2:
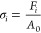
2where *F*_*i*_ is the tensile force imposed by each dead weight (index *i*) increment and *A*_0_ is the initial cross-section of the rod.^37^

### Poisson’s Ratio Measurements

**Figure 2:**
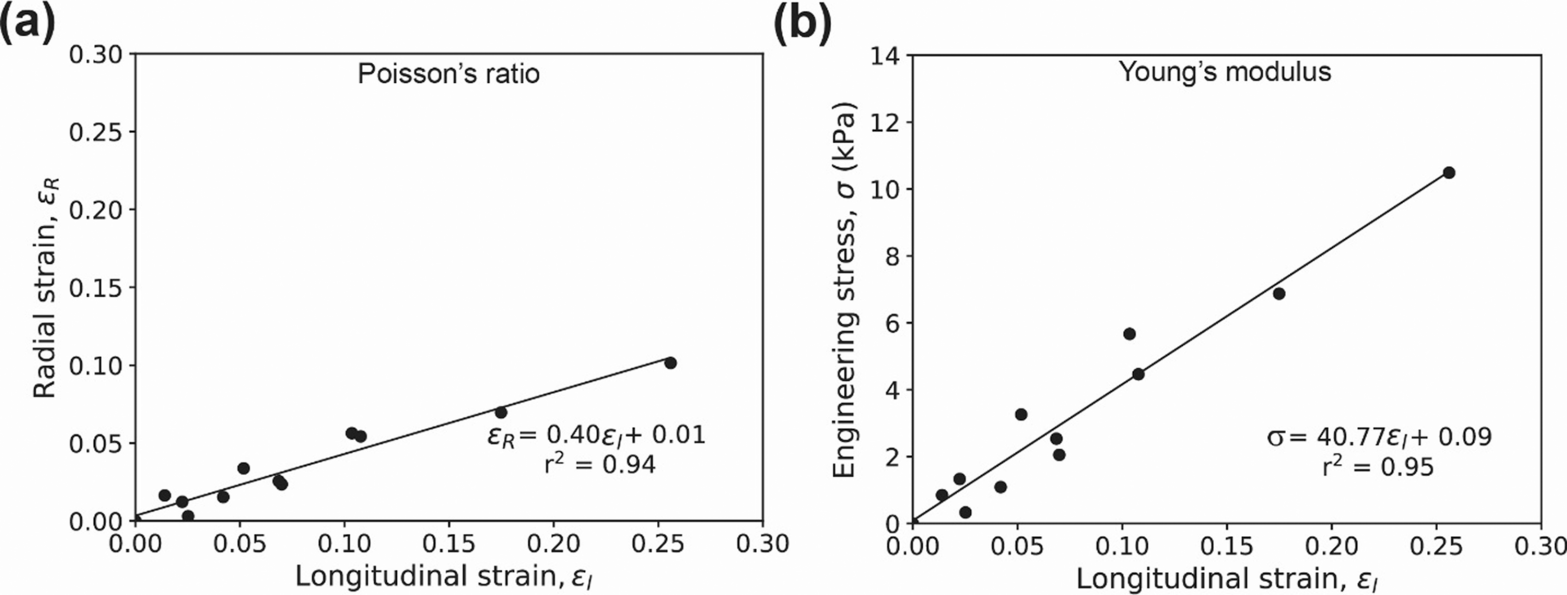
Representative scatter plots and linear regressions used to extract (a) Poisson’s ratio and (b) Young’s modulus values.

To calculate Poisson’s ratio ν, the radial strain ε_R_ was plotted as a function of the longitudinal strain ε_L_. A linear regression model was then used to calculate the slope, thus extracting the Poisson’s ratio ν = ε_R_/ε_L_, as illustrated in Figure 2a.

### Young’s Modulus Measurements

To calculate Young’s modulus *E*, the engineering strain *σ*_*i*_ was plotted as a function of the longitudinal strain *ε*_*L*_. A linear regression model was used to calculate the slope, and the modulus was evaluated using *E* = σ/ε_L_, as illustrated in Figure 2b. The maximum applied axial strains used to extract Young’s modulus *E* (using weights summarized in Table S1) were ε_L_ ≈ 0.25 for the three PAH gel formulations, which we call soft, intermediate, and stiff PAH gel, respectively. The strains measured before fracture were 0.31 ± 0.04, 0.57 ± 0.4, and 0.28 ± 0.03 for soft, intermediate, and stiff PAH gels, respectively (see Table S2). These maximum strains correspond to the last measurable strain before failure. The maximum applied strains used to extract Young’s modulus *E* were ε_L_ ≈ 0.9, 0.5, and 0.15 for 50:1, 20:1, and 10:1 for PDMS elastomer (using weights summarized in Table S3), respectively. The failure threshold was never reached for the PDMS elastomer samples.

A small prestress of 0.3 kPa was applied to PAH gel samples, 1.2 kPa to 50:1 PDMS elastomer samples, and 29 kPa to 20:1 and 10:1 PDMS elastomer samples, before starting to quantify strain values. This prestress resulted in a small extensional strain that stabilized the rod specimens. Typically, dog-bone-shaped specimens are recommended for uniaxial tests, to reduce the influence of stress concentrations induced by loading grips at each end of the specimen. Here, however, the specimen geometry was kept as simple as possible and linear strains were applied. The effects of the boundary at the center of the specimen were negligible as found experimentally and as shown in Figures S2 and S3. If the fiducial markers are placed between the center of the specimen and, in our measurements, at a maximum distance of 20% of the center line, Young’s moduli values are insensitive to fiducial marker location for both the PAH gels and the PDMS elastomer samples tested. However, this is not the case if the center of the specimen is not included in the measurement, as shown in Figure S2. The fiducial marker’s width must be large enough to generate a measurable intensity change. A horizontal line passing through the center of the specimen was chosen over two other geometries, a box of fixed 200-pixel width and a box of random width between the two fiducial markers to extract the intensity profiles, due to higher precision between independent measurements, as shown in Figure S3.

### Rheology

The PAH gel was created as described by Tse and Engler.^33^ Bulk shear rheological behaviors of the PAH gel and PDMS elastomer were characterized using a rheometer (Anton-Paar MCR-302e). The attachment geometry used was a sandblasted stainless-steel parallel plate (PP-25/S), 25 mm in diameter for all measurements. The roughness of the sandblasted surface prevented the slippage of the samples in contact with the steel surfaces. Rheology experiments for all gel samples were conducted at a temperature of 25 °C.

For PAH gel samples, a total of 510 μL of premixed PAH gel precursor solution (Table 1) was pipetted onto the bottom plate. Once in place, the sandblasted top parallel plate was slowly lowered until it reached a gap of 1.00 mm, ensuring that the gel sample bridged the top plate and filled the cavity without voids. PAH gel samples were then left to polymerize for 30 min.^33^ Once polymerization was complete, excess liquid was wiped and the gap was further lowered to 0.990 mm, corresponding to 1% compression strain in the axial direction. We find that the PAH gels cast between the parallel plates are very close to their fully swollen state, Figure S4.

For PDMS elastomer samples, a total of 2 mL of degassed and premixed base and curing agent solution (see Table 2) was cast onto a 35 mm Petri dish lid, resulting in a 1.5 ± 0.3 mm thick film after curing, measured with a vernier caliper. Discs were cut using a 25 mm stainless steel diameter hole punch to match the dimensions of the 25 mm sandblasted rheometer top spindle attachment. Before conducting measurements, the top spindle was lowered as the normal force measured by the rheometer was continuously monitored. We ensured that the normal force was slightly greater than zero (≈0.1 N) to ensure the spindle was in contact with the sample. The gap was then reduced to achieve a 1% compression strain in the axial direction. This prestrain combined with the sandblasted surface of the parallel plates avoids sample slippage and ensures contact during the shear tests.

The static shear modulus *G* may be obtained as the limiting value (in the limit of infinitesimally small frequency) of the measured storage modulus *G*′ measured in a frequency sweep experiment. To correspond with the definition of the shear modulus, these oscillatory experiments were conducted at small amplitudes corresponding to small strains. We performed a parallel, independent set of experiments in which the shear modulus was measured as a function of the shear strain γ. To also obtain an understanding of the range over which the material behaves as a linear elastic system, the samples were probed over a wide range of frequencies and strains. For the frequency sweep tests, the frequency ω varied between 0.1 and 10 rad/s (or equivalently 0.0159–1.59 Hz) at a constant shear strain γ of 1%. The value of the shear strain (1% shear strain) was chosen so that the material response was linear and facilitated the comparison of our results with published literature values.^33^ For the shear sweep experiments, the shear strain γ was varied between 0.1 and 10% at a constant frequency ω of 6.28 rad/s (or equivalently 1 Hz).

### Data Analysis and Statistics

Data analysis was performed using in-house python scripts (available for download at https://gitlab.com/randresen/facile-determination-of-the-poisson-s-ratio-and-young-s-modulus-of-polyacrylamide-gels-and-polydimethylsiloxane/-/tree/main/. In the plots shown, box and whisker plots indicate the median, with each individual data point corresponding to an independent measurement. Scatter plots show the mean and standard error of the mean.

## Results

### Static Tensile Poisson’s Ratio and Stiffness of the PAH Gel and PDMS Elastomer

**Figure 3:**
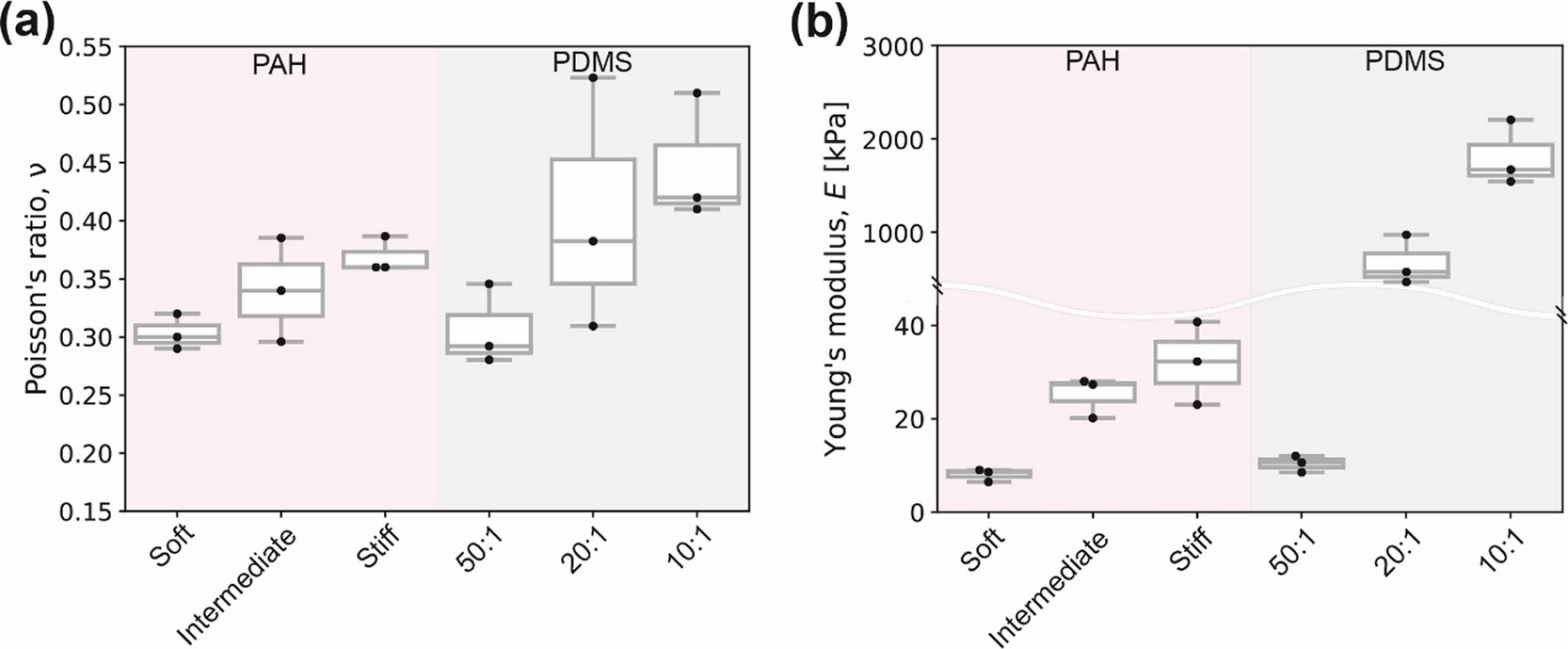
Elastic constants quantified via static tensile tests. (a) Poisson’s ratio values and (b) Young’s moduli of different PAH gel and PDMS elastomer formulations.

The elastic parameters measured from static tension tests for PAH gel and PDMS elastomer are shown in Figure 1 and other related details are summarized in Table S4. We observed that the Poisson’s ratio ν of both materials tested increased with increasing initial polymer volume fractions (for PAH gel) or cross-linking degree (for PAH gel and for PDMS elastomer), indicating that the gels were becoming less compressible, Figure 3a. For soft, intermediate, and stiff (expected from the fabrication protocol^6^) PAH gels, ν values were 0.30 ± 0.01, 0.34 ± 0.03, and 0.37 ± 0.01, respectively. For the 50:1, 20:1, and 10:1 PDMS elastomer, ν values were 0.31 ± 0.02, 0.41 ± 0.06, and 0.45 ± 0.03, respectively. The Young’s modulus *E* followed a similar qualitative trend as what we just described for the variation in ν of PAH gel and PDMS elastomer. Since PAH gels are fully swollen for static tension tests, these trends are consistent with classical polymer theory.^38^ As expected, and widely reported in the literature,^6,7,15^ Young’s modulus *E* of the PAH gel increased with increasing acrylamide concentration (polymer volume fraction), with *E* values ranging from 8.0 ± 0.8 to 25.2 ± 2.5 kPa, and 32.0 ± 5.1 kPa for soft, intermediate, and stiff PAH gel, respectively, Figure 3b. The results are in good agreement with *E* values obtained via AFM nanoindentation^6^ and other macroscopic studies.^27^

Increasing the curing agent content of PDMS elastomer results in a higher degree of cross-linking in the polymeric matrix and therefore increases *E*. The static tensile test *E* values measured for the PDMS elastomer were 10.4 ± 0.75, 667.4 ± 153.8, and 1802.0 ± 202.3 kPa for 50:1, 20:1, and 10:1 mixing ratios. These results are also in excellent agreement with other studies reporting on tensile characterization of PDMS elastomer using more sophisticated approaches, such as a universal testing machine or dynamic mechanical analysis.^9,39−41^

To ensure the elasticity of the PAH gel and PDMS elastomer, we quantified Young’s modulus during unloading, that is, by removing dead weights, and obtained very similar values for all conditions tested, Figure S5a,b and Table S5.

### Rheology of the PAH Gel and PDMS Elastomer

To characterize the shear modulus *G*′ of the soft, stiff, and intermediate PAH gel and of the 50:1, 20:1, and 10:1 PDMS elastomer, we conducted shear rheology measurements. Note that the zero-frequency shear modulus *G* may be obtained by examining the frequency dependence of the shear modulus *G*′ determined from rheology. As previously stated, we report *G* as the limit of *G*′ as the frequency ω tends to zero at small shear strain corresponding to the material being in the linear response regime. With this definition, the limiting value  is equivalent to the value ascertained from static shear tests. We compare then the equilibrium shear modulus *G* to the limit of *G*′ as the strain γ tends to zero  to quantify differences arising from frequency effects since the strain sweeps are conducted at a small but nonzero frequency.

**Figure 4:**
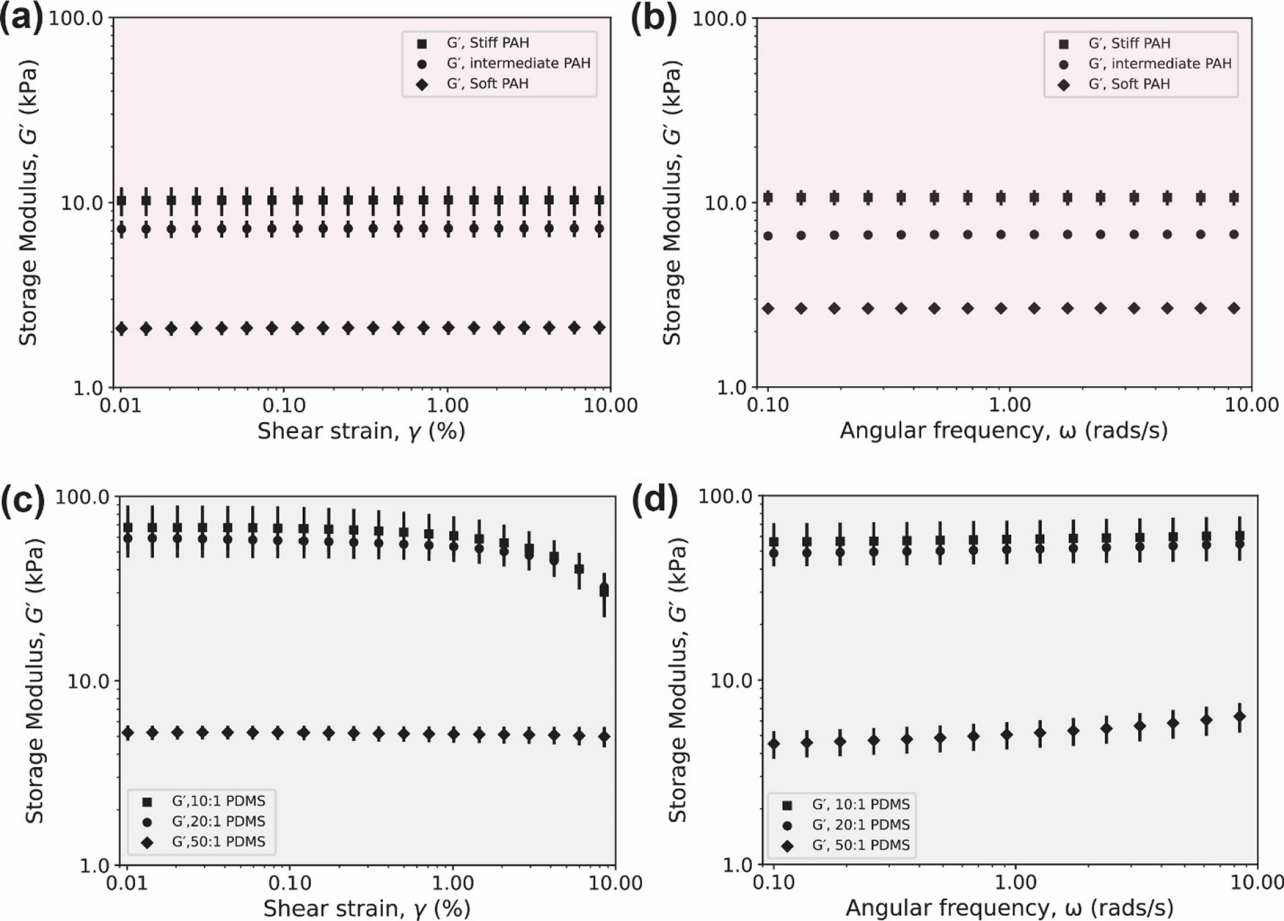
(a,c) Shear strain dependency of the dynamic storage modulus (*G*′) at a constant angular frequency of 6.28 rads/s (or 1 Hz). (b,d) Frequency dependency of the storage modulus (*G*′) at a constant shear strain of 1%. Three independent and new samples per condition are reported. Shown is the mean and the standard error of the mean.

We first present results for the PAH gel samples probed by rheology. Figure 4a shows the log–log curves of *G*′ of the soft, intermediate, and stiff PAH gel as a function of shear strain γ. Our results confirm that for small to moderately small shear strains (in the range of 0.01–10%), all PAH gel samples behaved as a linear elastic solid, as suggested by the near-constant (with negligible linear slope) values of *G*′. The average *G*′ values measured from the shear strain sweeps were 2.1 ± 0.2, 7.2 ± 0.8, and 10.7 ± 1.0 kPa, for soft, intermediate, and stiff PAH gel, respectively. Figure 4b shows the frequency dependence of *G*′. Our results reveal that for small to moderately small angular frequencies (in the range of 0.1–10 rads/s), the PAH gel behaves as a linear elastic solid. The measured *G*′ values for soft, intermediate, and stiff PAH gel measured from the frequency sweep tests were 2.7 ± 0.1, 6.6 ± 0.4, and 10.2 ± 1.1 kPa, respectively.

We further quantified the effect of compressive strain on the *G*′ of stiff PAH gel samples. As seen in Figure S6a,b and summarized in Table S6, variations in *G*′ are within experimental error and did minimally vary with increasing compressive strain.

We next present the results of our studies on the PDMS elastomer samples. Like PAH gels, we report *G* as the limit of *G*′ as the frequency ω tends to zero (), equivalent to a static shear test. We compare then the equilibrium shear modulus *G* to the limit of *G*′ as the strain γ tends to zero , to quantify differences in shear testing modes. Figure 4c shows the log–log curves of *G*′ as a function of shear strain γ (at a constant 1 Hz or 6.28 rad/s). For 20:1 and 10:1 PDMS elastomer samples, the linear elastic region indicates that the elastomer has predominantly linear elastic behavior up to a shear strain value γ of approximately 1%. Above 1% shear strain, 20:1 and 10:1 PDMS elastomer samples begin to soften. The average *G*′ values as a function of γ are 59.2 ± 4.6 and 67.9 kPa, respectively. However, the 50:1 PDMS elastomer behaved linearly over the full range of shear strains γ tested (up to 10%), with an average *G*′ value of 5.2 ± 0.3 kPa.

As opposed to the PAH gel, all three PDMS elastomer formulations showed different rheological responses between the sweep modes. Figure 4d shows the log–log curves of *G*′ as a function of the frequency ω. The 50:1 formulation shows stiffening. From an initial *G*′ value of 4.5 ± 0.5 kPa, *G*′ increased to a value of 6.4 ± 1.2 kPa, approximately 40% stiffer at the highest frequency tested. The 20:1 and 50:1 PDMS elastomer samples behaved differently. For these, *G*′ remained constant over the range of frequencies tested (up to 10 rad/s). We measured average *G*′ values of 48.7 ± 2.8 and 56.1 ± 8.5 kPa, respectively. Overall, the PDMS elastomer samples deviated from linearity, suggesting the importance of a priori knowledge of the application intended for the PDMS elastomer.

We further quantified the effect of precompression strain on *G*′, for both frequency (at constant 1% shear strain) and strain (at constant 1 Hz frequency) sweeps. As seen in Figure S6c,d, *G*′ increases with increasing compressive strain. This result emphasizes the importance of properly reporting characterization parameters, as the quantified values will vary significantly.

As mentioned previously, our first goal was to directly measure the Poisson’s ratio of the PAH gel and of the PDMS elastomer formulations. This provides a direct, macroscale measurement of the Poisson’s ratio from a static measurement. For a continuum linear elastic material deforming affinely, this geometric property also matches the Poisson ratio connecting *E* and *G* (the zero-frequency shear modulus). Thus, within this framework, the shear modulus estimated from the rheological measurements can be used to calculate *E*. Likewise, the *E* value from the static tension test may be used to estimate *G*. Our second goal was, therefore, to cross-evaluate and compare *E* obtained from the static tension test, with that estimated from shear rheology by using *G*. Specifically, we convert from Young’s modulus to the shear modulus by assuming the continuum relationship valid for a linear isotropic elastic material (eq 3) and using the experimentally determined value of the Poisson’s ratio,
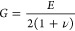
3

To reiterate, given that we treat the PAH gels and PDMS elastomers as linear isotropic elastic materials (and as observed experimentally by the tensile test and shear rheology), we assume that the geometrically determined Poisson’s ratio (ν = ε_R_/ε_L_) is equivalent to the Poisson’s ratio in eq 3.

### Comparison between Rheology and Static Tensile Tests of the PAH Gel

**Figure 5:**
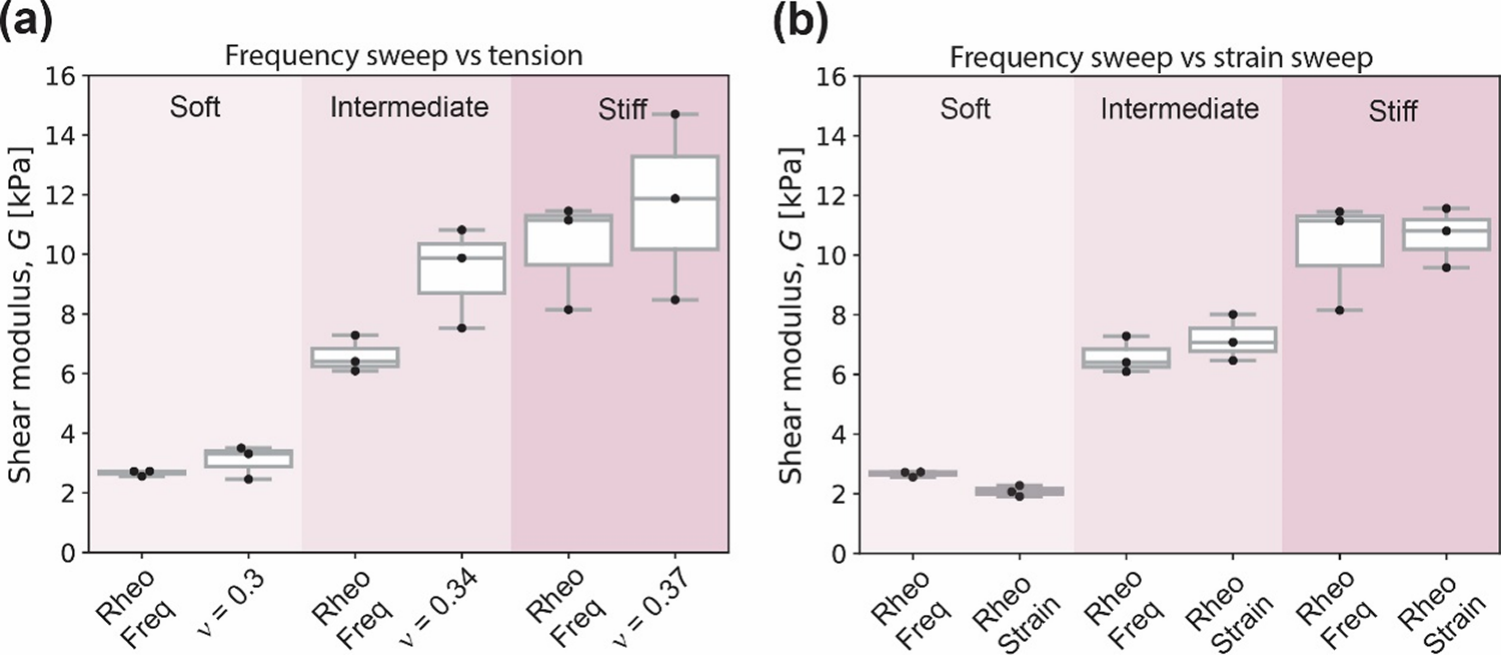
Cross-correlation of Young’s modulus (*E*) and shear modulus (*G*) (a) Comparison between *G* obtained from the zero frequency limit of the frequency sweep measurements (Rheo Freq, at constant 1% compressive strain) and shear modulus calculated using eq 3 for PAH samples, and (b) comparison between zero-frequency limiting value *G* (Rheo Freq) and shear sweep determined *G* (Rheo Strain, at 1 Hz) also at constant 1% compressive strain of PAH samples.

Most previous studies on PAH gel as substrates assume that it is incompressible with a Poisson’s value of 0.5. We consider the first measurable *G*′ value (i.e., at the lowest frequency) to be equivalent to the shear modulus *G* (). Figure 5a shows the cross-evaluation between the shear modulus deduced from *E* and ν measured from static tensile tests (labeled measured value) and the shear modulus measured from frequency sweep tests (labeled Rheo Freq). We observe that estimating *G* by using *E* and the Poisson ratio determined from the tension tests yields shear moduli *G* that are overall higher, with the ratio of shear moduli α having values of 0.86, 0.70, and 0.88 for soft, intermediate, and stiff PAH gels, respectively. We further compare the shear moduli *G* () at the fixed strain of 1% and *G* () at the fixed frequency of 1 Hz, and find that their ratio (defined as β = *G*_Freq_/*G*_Shear_) attains values 1.28, 0.92, and 0.96 for soft, intermediate, and stiff PAH gels, respectively, as summarized in Figure 5b. Note that β is a measure also of the modest effects of the nonzero but small frequency. The frequency value was chosen to compare with previously published literature values.

Overall, we observe small deviations (within experimental error) in the cross-correlation agreement between the rheology-determined shear modulus and our static tension-determined shear modulus.

We also note that while the assumption of incompressibility may be reasonable, this does not imply that it is a valid assumption as further discussed in the Discussion section and elsewhere.^13,15^ This is especially important when we consider dynamic effects specific to mechanobiology studies on cell motility on biomimetic hydrogel surfaces. For instance, the deformation fields induced in the substrate due to the contractile and time-dependent stresses in focal adhesion regions depend crucially on Poisson’s ratio.^29,30,42^ A second instance where Poisson’s ratio may play an important role is in the rate-dependent deformation of initially planar hydrogels of finite stiffness. Here, axial and normal deformations are coupled via Poisson’s ratio and together determine the time-dependent forces felt by the indenter. Such rate-dependent deformations are not static and also involve significant poroelastic effects in fluid-infiltrated hydrogels and gel-like materials.^43^

### Comparison between Rheology and Static Tensile Tests of the PDMS Elastomer

**Figure 6:**
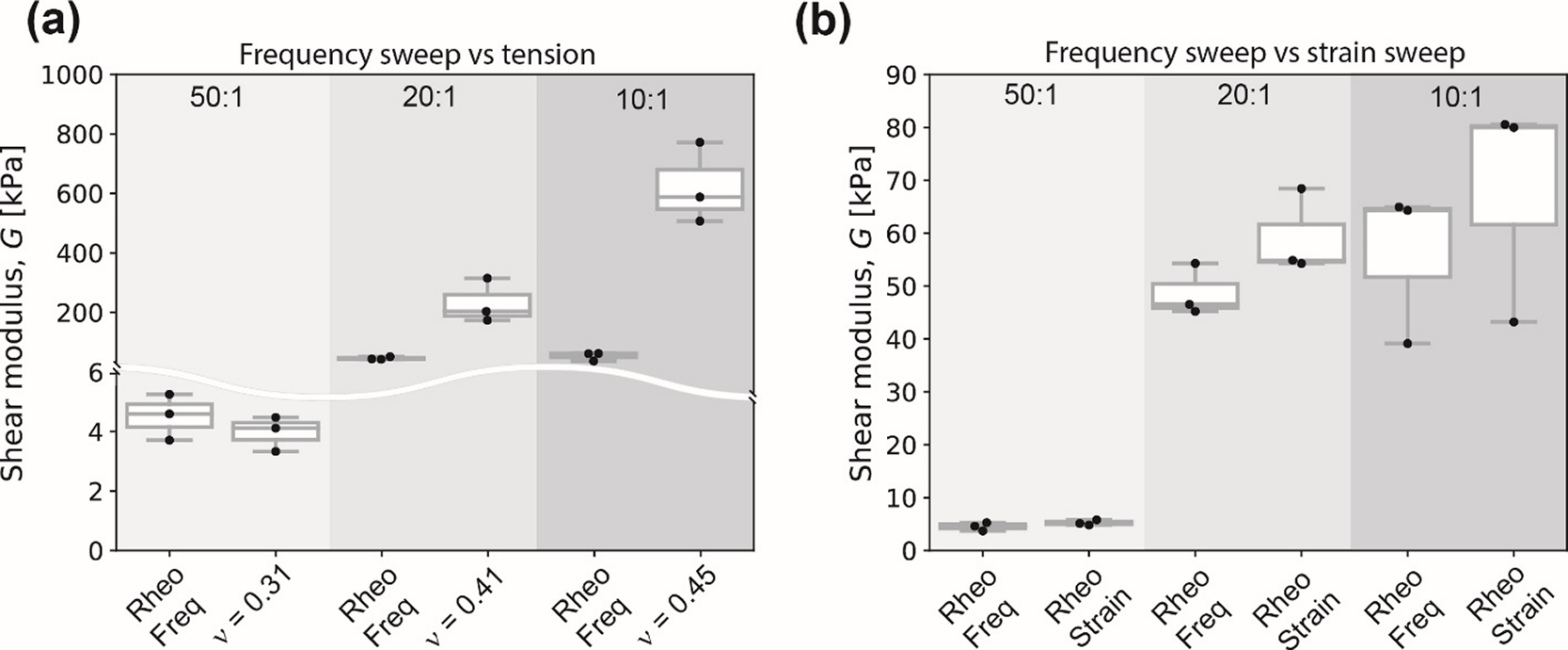
Cross-correlation of Young’s modulus (*E*) and shear modulus (*G*) as a function of Poisson’s ratio (ν). (a) Comparison between shear strain sweeps at a constant ω of 1 rad/s. (b) Comparison between frequency sweeps at a constant 1% compressive strain of PDMS samples. Note that the *y*-axes have a range break.

Next, we extended the above-described approach to analyze the cross-correlation of elastic constants obtained between static tensile tests and shear rheology for PDMS elastomer rods. We asked whether the cross-correlation of the two bulk characterization modes (tension vs. compression with shear) was in good agreement, as seen for the PAH gel. Figure 6a shows the cross-evaluation between *G* measured from frequency sweep tests and calculated using Young's moduli and Poisson’s ratio values extracted from static tensile tests. For PDMS elastomers, we observe that the tension obtained values yield shear moduli *G* are in good agreement for the 50:1 samples, and considerably higher for 20:1 and 10:1, with the relative shear ratio (defined as α = *G*_Rheo_/*G*_Tension_) values of 1.13, 0.21, and 0.09 for 50:1, 20:1, and 10:1 PDMS elastomers, respectively. We further compared the shear moduli *G* () and *G* (), and find that their ratio (β = *G*_Freq_/*G*_Shear_) has values 1.16, 1.21, and 1.21 for 50:1, 20:1 and 10:1 PDMS elastomers, respectively, as summarized in Figure 6b.

## Discussion

**Figure 7:**
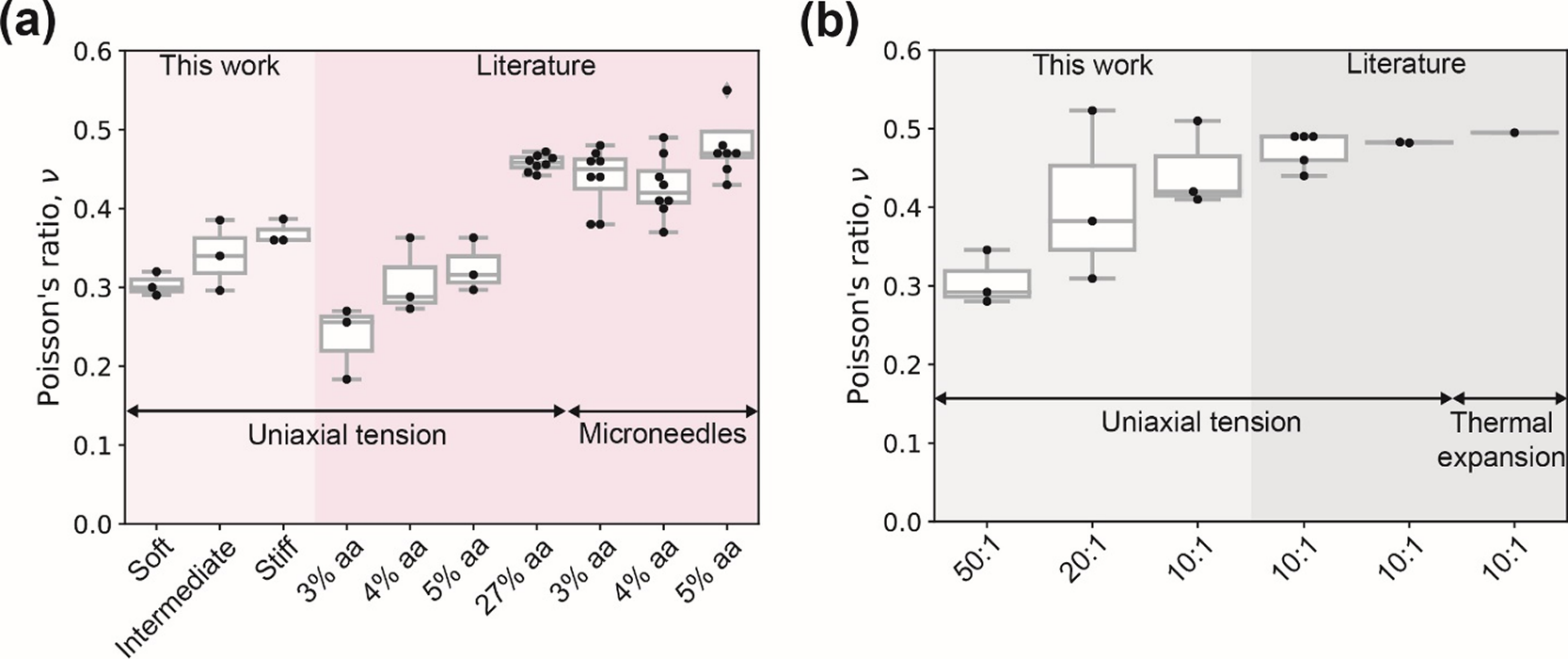
Comparison of Poisson’s ratio ν values from this work and other works for (a) PAH gels and (b) PDMS elastomers. All PDMS elastomer values reported correspond to the Sylgard 184.

The primary goal of this work was to evaluate Poisson’s ratio of gels and polymers such as PAH gel and PDMS elastomer relevant to a range of disciplines, including mechanobiology, bioengineering, and biomaterials, among others. Many experimental studies that interpret data obtained using these gels and elastomers as substrates, as well as other computational studies assume that the PAH gel is incompressible and thus ν = 0.5. Here, we corroborate that this assumption is not correct and that ν of PAH gels increases with increasing polymer volume fraction and cross-linking degree, as previously reported^15^ consistent with theoretical scalings derived for networked polymeric systems.^20,44^ The static tensile test described here obtained bulk properties. Local elastic properties can be length-scale dependent. For instance, approaches such as embedded microneedles actuated with external magnetic fields yield different values, emphasizing the importance of the scale of the characterization.^45^ Our ν values for PAH gel and others^14,15,45^ are summarized in Figure 7a. We observe a similar behavior for PDMS elastomer, that is, ν increased with increasing degree of cross-links. Our ν values for PDMS elastomer and others^16,17,46^ are summarized in Figure 7b.

Quantifying these two elastic constants is crucial for experimental platforms, such as traction force microscopy (TFM), the most widely employed contractile force measurement approach for adherent cells, which relies on knowledge of the force–displacement relationship and mechanical properties of the substrate.^1,2,47−51^ In a recent study, Javanmardi et al. demonstrated that applying the correct ν is crucial for accurate force reconstruction employing TFM approaches.^15^ One advantage of our approach is that it is extremely simple. Specifically, a single experiment can be used to extract values of both Young’s modulus *E* and Poisson’s ratio ν, for soft materials relevant to mechanobiology. However, careful attention needs to be paid to ensure that the measured values are accurate. Two crucial aspects need to be considered: (i) the fiducial markers must be placed at equal distances from the geometric center of the free specimen length (Figure S2) for accurate Young’s modulus, and (ii) the profile line must be traced horizontally through the geometric center (Figure S3) for accurate Poisson’s ratio determination.

Measurement uncertainties can come from various sources. In our measurements, the highest resolution was with a pixel-to-micron ratio of approximately 6 μm/px. This value results from the camera, lens, working distance, alignment, and illumination. For the case of radial deformations, the limiting factor is the width of the specimen edge detected, approximately 10–30 px, that is, 60–180 μm. Radial deformations must displace the intensity peak position above a confidence interval, for example, half of the width of the measured specimen edge width. The fiducial markers’ sensitivity is analogous to the radial deformations, and the smaller the marker, the higher the resolution. In our measurements, applied weights for PAH gels in increments of 2 g for soft and 5 g for intermediate and stiff PAH gels, and for PDMS elastomers in increments of 5 g for 50:1, 20:1200, and 10:1300 g generated large enough radial and longitudinal deformations to displace the peak positions.

Hydrogel networks due to their various cross-linking properties, and the fluid (water or biological fluid) permeating them have both viscoelastic and viscoplastic properties. Two main types of cross-links used in formulating the hydrogels are chemical cross-links and physical cross-links which both confer structure and elastic properties but contribute differently in terms of both frequency-dependent and static properties. Chemical cross-links, for instance, in PAH gel due to irreversible chemical bonding provide greater rigidity and enable static prestressed states. Physical cross-links are comparatively weak and may be temporary, allowing for the relaxation of imposed stresses. During the deformation of PAH gel, hydrogen bonds may form and break depending on how close different polymeric chains get. Additionally, other temporary physical cross-links, such as chain entanglements, confer some strength to the hydrogel; they may slip and unentangle under stress. Transient cross-links may significantly contribute to dissipation (and thus to the viscous loss modulus) when hydrogels are subjected to frequency-dependent deformations. Finally, hydrogels are permeated by fluid; at equilibrium conditions, they are under mechanically balanced conditions. In the nearly (fully) swollen state, as is the case for our samples in independent characterization approaches, static tension tests, and shear rheology, the osmotic pressure in the fluid-filled pores supports the polymer network/scaffold and provides means to support and balance externally imposed stresses.

Usually, the analyses of the elastic response of materials such as metals assume that the material's response is similar and symmetric for small tension and compression strains.^52^ This is not the case for soft polymeric networks, in which tension-compression asymmetry (TCA) is commonly found.^53^ For instance, Young’s modulus of hydrogels and elastomers evaluated from tension measurements can differ from compression measurements by orders of magnitude.^53^ While both PAH gel and PDMS elastomer have chains bridged by chemical and physical bonds, particularly at low cross-linking densities, PAH gel (a hydrogel) consists of loosely cross-linked networks permeated by water molecules, and PDMS elastomer consists of chains that are more severely restricted due to increased cross-linking. This difference tremendously impacts the molecular mechanisms affecting network responses to stress. Furthermore, when fully swollen hydrogels are subject to deformations that impact local volume conservation and pore deformations, such as compression or tension, water is forced to flow through the pores. The permeability of the network enabling this flow is controlled by the size of the mesh, denoted by ξ. If ξ becomes small, as is the case for our PAH gel, Table S7, the osmotic pressure and hence the shear modulus (or equivalently, Young’s modulus) increases. Classical theories due to Flory^54^ and de Gennes^20^ relate shear modulus *G* (related to polymer concentration), and osmotic pressure Π, via the relationship *G* ≈ Π = *k*_B_*T*/ξ^3^,^20^ where *k*_B_ is Boltzmann constant, *T* the absolute temperature, and ξ the mesh size, which is a linear measure of the free space among polymer chains. In summary, the mechanical response, be it compression, shear, or tension of PAH gel, will be strongly coupled to ξ.^44^ We note that the osmotic pressure may also be written in terms of the concentration of polymer and correlated with the mesh size via concepts invoking the Kuhn length and radius of gyration of molecules under various swelling conditions.

**Figure 8:**
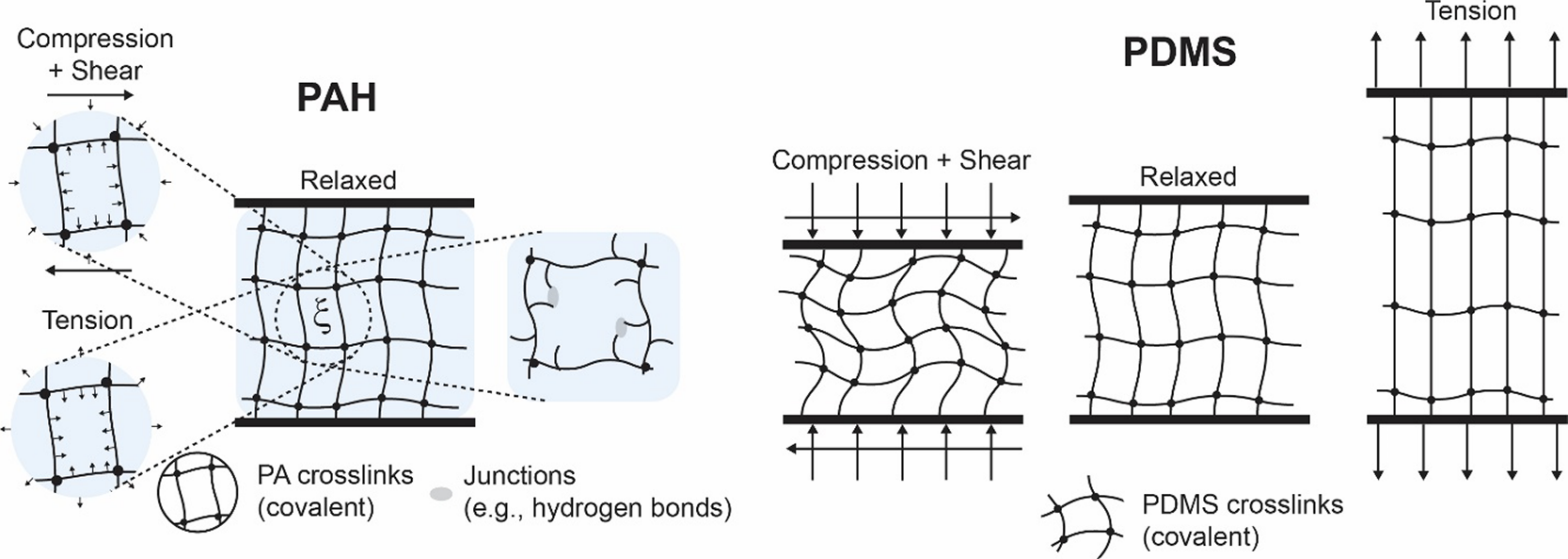
Schematic figures illustrating how osmotic pressure in PAH hydrogels, mesh size, and the presence of weaker junctions (physical cross-links) mediate mechanical response (left). Figures on the right show the change in network state (with links dangling/buckled under compression, or fully stretched under tension) for a densely cross-linked (10:1, 20:1) PDMS.

Under tension or compression and for small perturbations (i.e., small strains or small frequencies), fluid is forced through the porous, elastic networks characterized by the mesh size ξ. We note from our experiments that static tension tests and shear rheology measurements for PAH gel show good agreement. Richbourg et al. cross-correlated five independent stiffness measurement methods for various poly(vinyl alcohol) (PVA) hydrogels, also finding excellent agreement.^55^ This suggests that for relatively simple hydrogels, that is, hydrogels composed of monomers with minimal side group bulkiness, such as PAH gel (i.e., acetamide) or PVA (i.e., hydroxy), osmotic pressure could be seen as the main mechanism governing the mechanical response. Contrarily, PDMS elastomer, particularly at 10:1 or 20:1 base-to-curing ratios, where the network is heavily cross-linked and molecular linkages restricted, and junctions/cross-links remain “active” during tension contributing to most of the extensional stress. However, the situation changes under compression and the buckling of polymeric chains causes local softening. In other words, buckling of the junctions causes the polymeric chains to switch to a dangling state, implying that the stresses in the involved chains are reduced significantly. While this hypothesis remains to be tested more carefully, the mechanism described, schematized in Figure 8, explains the experimental observations for PDMS elastomer, in which 10:1 samples (with the highest cross-linking degree) show a high level of discrepancy between rheology (small compression plus shear) and static tension tests. The discrepancy decreases for 20:1 and even more so for 50:1 PDMS elastomer samples, consistent with a decrease in the degree of cross-linking and resembling the hydrogel mode (without the osmotic pressure contribution). While we identified that there is a 14% fraction of oligomers that did not polymerize into the network for the 50:1 PDMS elastomer samples, solvent migration would be minimal and has been shown to not have a significant contribution to the elastic constants of PDMS elastomers.^36^

We conclude the discussion by summarizing how extensions of classical poroelasticity theories by Biot allow a description of shear modulus *G*, and Poisson ratio ν in terms of the initial preparation state of the gel, its dry state properties including cross-linking, and the swelling ratio. Hydrogels are viscoelastic, and thus the elastic moduli as well as the Poisson ratio are functions of the probing frequency. Hydrogels are also poroelastic,^56−59^ with the flow of water and boundary conditions (whether the sample is surrounded by water, jacketed, or next to a rigid surface) affecting the measured moduli.^59^ This suggests that the elastic properties of swollen gels under deformation may be interpreted using the poroelastic theory. Indeed, Hu et al.^58,60^ present a small deformation poroelastic theory for swollen gels by linearizing the equations of nonlinear elasticity theory about an isotropically swollen state. In their model, the dry state is subject to swelling in a solvent (water). It is assumed that the polymer gel is stress-free and isotropically swollen at this initial reference state. Furthermore, it is also assumed that the volume of the gel changes only by solvent absorption/desorption. The swollen gel is then subject to a small strain deformation, and equations are derived. For isotropic swelling, the swelling ratio (relative to the dry state), λ_R_ is the same in all directions. Linearizing the expressions for the chemical potential and nominal stress obtained from the Flory–Rehner theory,^54,56^ Hu et al. derive expressions for the Lagrange multiplier (overall pore pressure and the osmotic pressure), the chemical potentials μ and μ_R_ in the reference and deformed states, the Cauchy stress written using Einstein notation σ_*ij*_, the shear modulus *G*, the network permeability *k*, and the Poisson’s ratio ν that we list below (eqs 4–7):
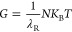
4
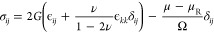
5
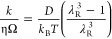
6
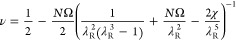
7

In eqs 4–7, *C* is the nominal solvent concentration (number of solvent molecules per unit volume of polymer), *N* is the effective number of polymer chains per unit volume of the polymer, χ is the Flory parameter for the interaction between the solvent and the polymer, Ω is the volume per solvent molecule, and *T* is the absolute temperature. The term (μ – μ_R_)/Ω may be identified with the pore pressure in the Biot theory and connects the mechanical description of pressure as a Lagrange multiplier to enforce the incompressibility of the molecular constituents to a description based on chemical potentials. The permeability is related to the mesh size introduced earlier. While we cannot directly relate eq 7 to our measured Poisson ratios, the overall picture of swelling decreasing the Poisson ratio substantially from its incompressible value is consistent with our measurements. Specifically, in the dry state, the Poisson ratio is 1/2. Swelling decreases the shear modulus *G* and decreases the Poisson ratio progressively relative to that of the dry state. Collectively, the findings presented herein underscore the significance of defining the elastic constants, particularly the Poisson’s ratio, in addition to Young’s modulus or shear modulus of soft materials, such as PAH gels and PDMS elastomers, instead of relying on the assumption of incompressibility.

## Conclusions

In this study, we characterized the bulk mechanical responses of the PAH gel and PDMS elastomer by varying the network compositions. Specifically, we quantified the Poisson’s ratio ν and Young’s modulus *E* via static tension tests. We show that the Poisson’s ratio varies from the value for incompressible materials (ν = 0.5) and that its value depends on the cross-linking degree (or alternatively, mesh size). Furthermore, we performed shear rheology to obtain the shear modulus *G* of PAH gel and PDMS elastomer and found that for the PAH gel, the cross-correlation of the elastic constants obtained from the independent methods is in very good agreement but not for PDMS elastomer. Together, our study emphasizes the importance of accurately characterizing *E* and ν of gels and elastomers rather than assuming the incompressible value, especially for use in mechanobiology studies. Our method provides an easy, accessible, and affordable means to achieve this characterization using materials and means commonly found in most laboratories.

## Supporting Information Available

Gel fraction of elastomers, static tensile test dead weights used, swelling ratio of PAH gels, gel fraction determination of PDMS elastomers, strain quantification and intensity profile analyzer software (IPA), swelling of PAH gels, elasticity constants, validation of elasticity, and effects of prestrain on bulk rheology measurements (PDF)

## Abbreviations

PAH gel: polyacrylamide hydrogel
PDMS elastomer: polydimethylsiloxane elastomer
AFM: atomic force microscopy
TFM: traction force microscopy
TCA: tension-compression asymmetry
PVA: poly(vinyl alcohol)
