# Supplementary material for: Facile Determination of the Poisson’s Ratio and Young’s Modulus of Polyacrylamide Gels and Polydimethylsiloxane
Source: ACS Appl Polym Mater. 2024 Feb 5;6(4):2405–16. doi: 10.1021/acsapm.3c03154 (PMC10897882; doi:10.1021/acsapm.3c03154)
Supplement: Supplementary file 1 — ap3c03154_si_001.pdf [file ap3c03154_si_001.pdf]

Supporting Information

Facile determination of the Poisson's ratio and Young's modulus of polyacrylamide gels and polydimethylsiloxane

Ariell Marie Smith[a‡,] *Dominique Gabriele Inocencio*[a‡], *Brandon Michael Pardi*[a], *Arvind Gopinath*[b,c,*], *and Roberto Carlos Andresen Eguiluz*[a,c*]

[a]Department of Materials Science and Engineering, School of Engineering, University of California, Merced, 5200 N. Lake Rd., Merced, CA 95344, United States of America.

[b]Department of Bioengineering, School of Engineering, University of California, Merced, 5200 N. Lake Rd., Merced, CA 95344, United States of America.

[c]Health Sciences Research Institute, University of California Merced, Merced, 5200 N. Lake Rd., Merced, CA 95344, United States of America.

Corresponding authors:

*randreseneguiluz@ucmerced.edu

*agopinath@ucmerced.edu

‡ AMS and DGI contributed equally to this paper

KEYWORDS polyacrylamide hydrogel, polydimethylsiloxane, Poisson's ratio, Young's modulus, shear rheology

Figure S1. PDMS elastomer gel fraction
Table S1. Dead weights used for static tension tests of PAH gels
Table S2. Weights and strains applied before fracture of PAH gels
Table S3. Dead weights used for static tension tests of PDMS elastomers
Figure S2. Sensitivity measurements of fiducial marker spacing and location
Figure S3. Sensitivity measurements of intensity profile on Poisson's ratio
Figure S4. Mass and swelling ratio of PAH gels.
Table S4. Elastic constant values obtained via static tension and rheology of PAH gels and PDMS elastomers
Figure S5. Loading and unloading values of Young's modulus confirming PAH gels and PDMS elastomer reversible elasticity
Table S5. Average loading and unloading values of Young's modulus confirming PAH gels and PDMS elastomer reversible elasticity
Table S6. Effect of pre-compression strain on shear moduli of PAH gels and PDMS elastomers obtained via bulk shear rheology
Figure S6. Plots of pre-compression strain on shear moduli of PAH gels and PDMS elastomers obtained via bulk shear rheology
Table S7. Estimated mesh sizes obtained from $G_{\text{FrequencySweep}}$

Gel fraction of PDMS elastomers

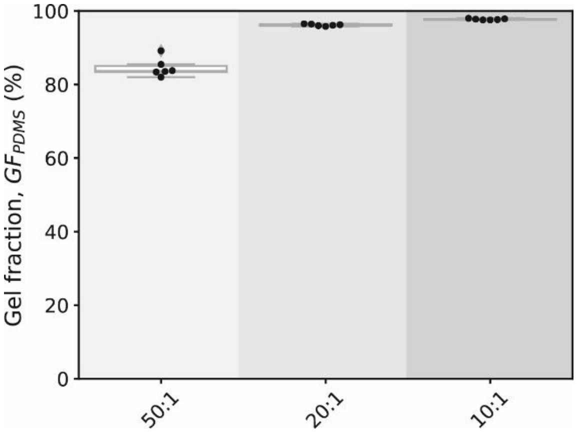

Figure S1. PDMS elastomer gel fraction obtained after extracting oligomers with chloroform for 50:1, 20:1, and 10:1 samples.

Static tensile test dead weights

For static tension tests, a series of dead weights from a calibration weights set were added (during the loading stage) or removed (during the unloading stage) to the PAH rods (see SI Table 1). In SI Table 2, we list the maximum weights and strains measured before loading of the subsequent weight that led to specimen fracture. The dead weights used for PDMS rods are listed in SI Table 3. The table summarizes the total dead weight at each weight increment.

Table S1. Dead weights used for static tension tests of PAH.

**Stiff PAH**	**Intermediate PAH**	**Soft PAH**	**Weight, *W* (g)**
Pre-stress	Pre-stress	Pre-stress	1.22
Measurement 1	Measurement 1	Measurement 1	0.69
Measurement 2	Measurement 2	Measurement 2	1.38
Measurement 3	Measurement 3	Measurement 3	3.53
Measurement 4			5.53
Measurement 5	Measurement 4	Measurement 4	8.53
Measurement 6	Measurement 5	Measurement 5	13.53
Measurement 7	Measurement 6	Measurement 6	18.53
Measurement 8	Measurement 7	Measurement 7	23.53
Measurement 9	Measurement 8	Measurement 8	28.53

Table S2. Weights and strains applied before fracture.

**Sample**	**Weight before fracture, $W_{\text{Before Failure}}$ (g)**	**Strain before fracture, $\varepsilon_{\text{Before Failure}}$**
Stiff PAH	55.0 ± 8.7	0.28 ± 0.03
Intermediate PAH	51.7 ± 22.5	0.57 ± 0.4
Soft PAH	14.3 ± 0.6	0.31 ± 0.04

Table S3. Dead weights used for static tension tests of PDMS.

**10:1 PDMS**	**20:1 PDMS**	**Weight, *W* (g)**		**50:1 PDMS**	**Weight, *W* (g)**
Pre-stress	Pre-stress	102.6		Pre-stress	4.6
Measurement 1	Measurement 1	200		Measurement 1	5
Measurement 2	Measurement 2	500		Measurement 2	10
Measurement 3	Measurement 3	700		Measurement 3	15
Measurement 4	Measurement 4	900		Measurement 4	20
				Measurement 5	25

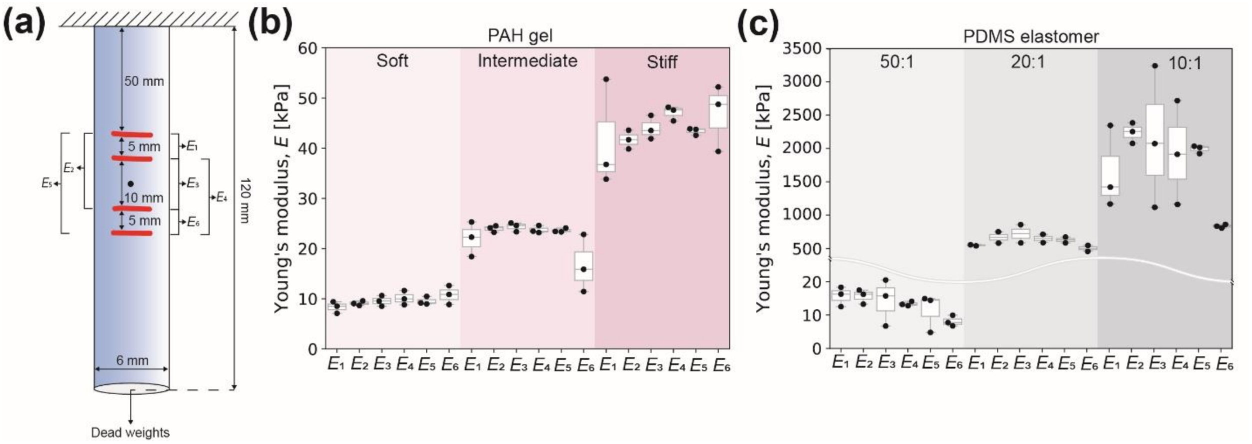

Figure S2. Sensitivity measurements of fiducial marker spacing and location, and their effects on measured values of the Young's modulus. (a) Schematic indicating the location of fiducial markers along tensile specimens. Values of the Young's modulus were obtained from various fiducial marker combinations for (b) PAH gels and (c) PDMS elastomers.

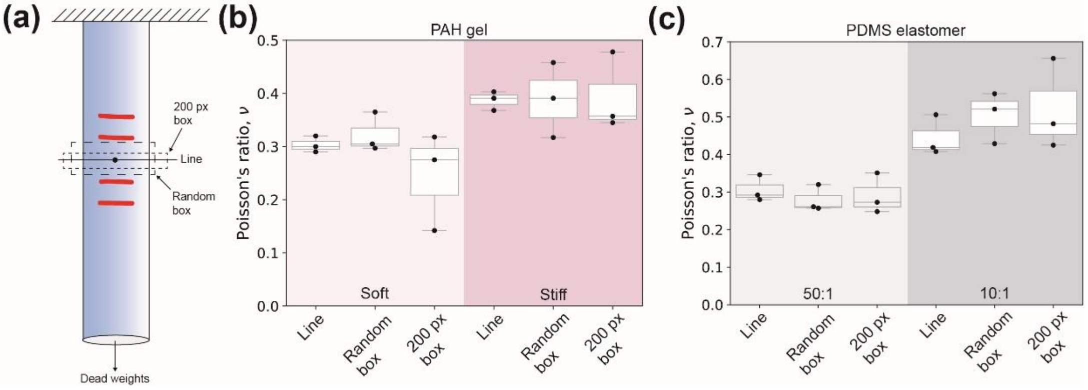

Figure S3. Sensitivity measurements of intensity profile on Poisson's ratio. (a) Schematic indicating the geometry used to extract the radial intensity profiles: line profile, box of fixed width (200 pixels(px)), and box with random width. Poisson's ratio values were calculated for the three different approaches to extract the intensity profiles (and thus the radial strain) for (b) PAH gels and (c) PDMS elastomers.

Swelling ratio of PAH gels

Soft, intermediate, and stiff PAH samples were allowed to polymerize in 6 mm diameter disposable straws for 30 minutes as described in the PAH rod sample preparation section. Each sample was cut into multiple

sections 12.7 mm in length. Samples were taken from the top, middle, and bottom sections of the rod. To measure the weight, change due to water intake, each sample was weighted directly after polymerization (*i.e.*, as cast) and placed in milliQ water. Each sample was weighed at 8 hrs. We use the mass change to define the swelling ratio (SR_{PAH}), calculated using eq. SI 1 using data from two time points.

$$SR_{PAH} = \frac{(W_{swollen} - W_{as\ cast})}{W_{as\ cast}} * 100 \qquad \text{(SI 1)}$$

where SR_{PAH} is the swelling ratio of the PAH gel sample, $W_{swollen}$ is the weight of the PAH gel at either 12 hrs or 24 hrs incubation in milliQ water, and $W_{as\ cast}$ is the weight of the PAH gel after fully polymerized.

Gel fraction determination of PDMS elastomers

PDMS elastomer gel fraction was quantified for 50:1, 20:1, and 10:1 samples. Three samples were prepared using the method described in the main text. Three pieces of different lengths were cut from each stiffness and weighed before being immersed in chloroform, used as the extraction solvent.[1] The extraction was performed for 5 hours, followed by an ethanol bath for 20 minutes. Extracted and swollen PDMS elastomer pieces were then placed in an oven for 19 hrs. at 60 °C. and weighed again. Gel fraction was determined using eq. SI 2

$$GF_{PDMS} = \frac{(W_{swollen} - W_{extracted})}{W_{as\ cast}} * 100 \qquad \text{(SI 2)}$$

where GF_{PDMS} is the gel fraction of the PDMS elastomer sample, $W_{as\ cast}$ is the weight of the PDMS elastomer as cast, and $W_{extracted}$ is the weight of the PDMS elastomer after the extraction process.

Strain quantification and Intensity Profile Analyzer software (IPA)

To quantify the PAH and PDSM dimensional changes in pixels, a digital single-lens reflex camera (Nikon, D750) with a macro lens (Nikon, AF-S Micro Nikkor 105) mounted on a tripod to take pictures via a wireless intervalometer to prevent mechanical drift. The PAH or PDMS rods were imaged once with each incremental step of dead weights added, ensuring that the fiducial markers were within the field of view. The camera remained static. Images were subsequently post-processed in FIJI (NIH). A horizontal line for diameter quantification or a vertical line passing through the center of the fiducial markers were traced, followed by the Plot Profile function under the Analyze menu of FIJI. The pixel and intensity values were saved in excel file format, and further analyzed using the using an in-house Python based tool. The tool, named call Intensity Profile Analyzer (IPA) is available for download at:

https://gitlab.com/randresen/facile-determination-of-the-poisson-s-ratio-and-young-s-modulus-of-polyacrylamide-gels-and-polydimethylsiloxane/-/tree/main/

The "Readme", describing the execution sequence and content is available as a separate file together with the main source code, as well as a sample spread sheet file.

Swelling of PAH gels

To determine if the PAH samples polymerized between the parallel plates of the shear rheometer were fully swollen, we quantified the water uptake after polymerization after 8 hrs fully immersed in milliQ water. Soft, intermediate, and stiff PAH resulted in a swelling ratio of 1525 ± 76 %, 970 ± 25 %, and 910 ± 51 % for as cast samples relative to dry and 1580 ± 83 %, 1143 ± 20 %, and 933 ± 5 % for fully swollen samples relative to dry samples, indicating the as-cast samples are close to fully swollen directly after polymerization.

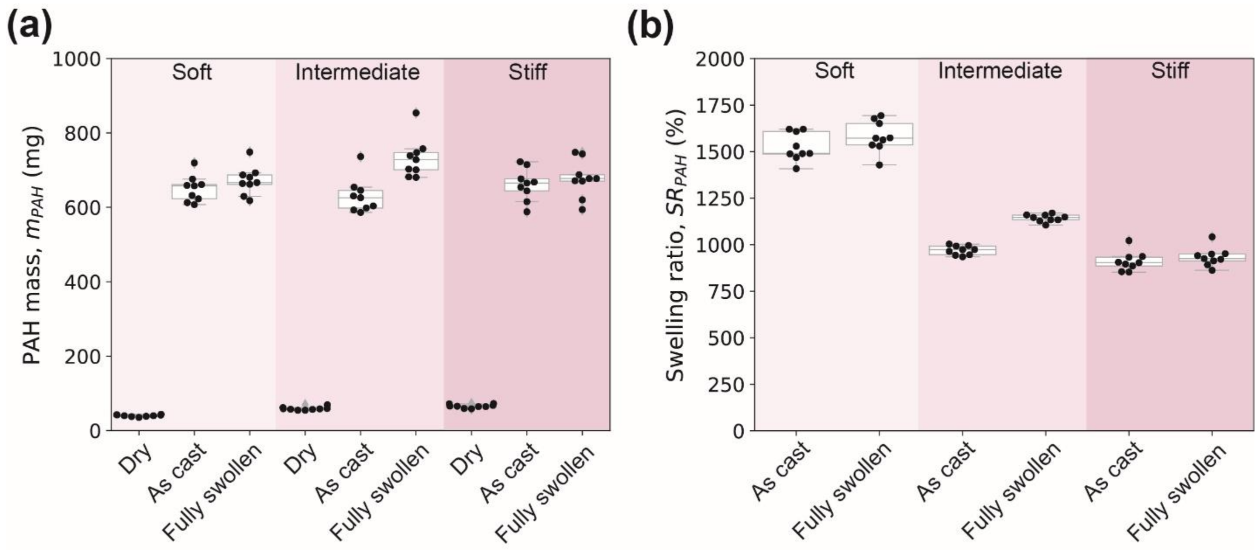

Figure S4. (a) PAH gel masses for dried samples, as cast, and fully swollen, used to calculate (b) the swelling ratios for as cast and fully swollen conditions relative to dry samples.

Elastic constants

A summary of the elastic constants for PAH and PDMS samples obtained via static tension tests and rheology is presented in SI Table 4.

Table S4. Elastic constants obtained from the static tension tests and shear rheology measurements with 1 % compression strain. $G'_{FrequencySweep}$ values are obtained from the frequency sweeps, taking the asymptotic zero-frequency limit. $G'_{StrainSweep}$ values are obtained from the strain sweeps, taking the zero-strain limit.

Sample	Poisson's ratio, ν	Young's modulus, $E_{Tension}$ (kPa)	Shear modulus, $G'_{FrequencySweep}$ (kPa)	Shear modulus, $G'_{StrainSweep}$ (kPa)
Soft PAH	0.30 ± 0.01	8.0 ± 0.8	2.7 ± 0.1	2.1 ± 0.2
Intermediate PAH	0.34 ± 0.03	25.2 ± 2.5	6.6 ± 0.4	7.2 ± 0.8
Stiff PAH	0.37 ± 0.01	32.0 ± 5.1	10.2 ± 1.1	10.7 ± 1.0
50:1 PDMS	0.31 ± 0.02	10.4 ± 0.75	4.5 ± 0.5	5.2 ± 0.3
20:1 PDMS	0.41 ± 0.06	667.4 ± 153.8	48.7 ± 2.8	59.2 ± 4.6
10:1 PDMS	0.45 ± 0.03	1802.0 ± 202.3	56.1 ± 8.5	67.9 ± 12.4

Validation of elasticity

We extracted the value of the elastic (Young's) modulus, E, during the loading and unloading of dead weights to confirm that the tensile deformations were within the elastic reversible regime, as shown in SI Figure 1. The numerical values of the moduli extracted from experiments are summarized in SI Table 4.

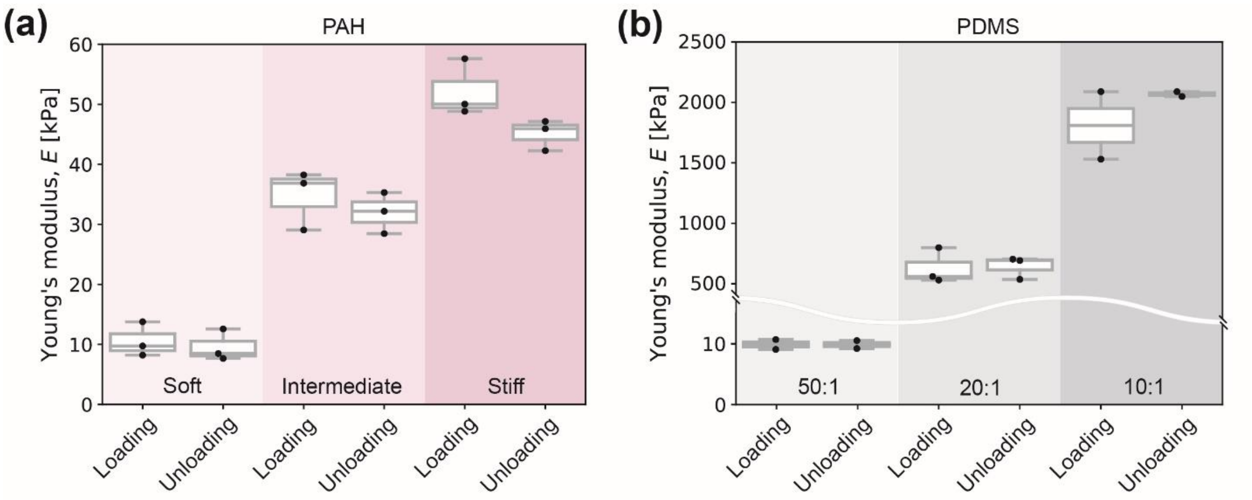

Figure S5. Young's modulus values comparison obtained from loading and unloading to confirm elasticity for (a) PAH and (b) PDMS.

Table S5. Average of loading and unloading for both PAH and PDMS

**Sample**	**Young's modulus, $E_{Loading}$ (kPa)**	**Young's modulus, $E_{Unloading}$ (kPa)**
Soft PAH	10.58 ± 2.87	9.58 ± 2.63
Intermediate PAH	34.72 ± 4.95	31.98 ± 3.43
Stiff PAH	52.16 ± 4.76	45.11 ± 2.54
50:1 PDMS	9.93 ± 1.16	9.91 ± 0.93
20:1 PDMS	622.37 ± 147.07	636.61 ± 93.61
10:1 PDMS	1802.48 ± 395.17	2061.86 ± 28.31

Effects of pre-strain on bulk rheology measurements

To evaluate rheological response under compression for both stiff PAH and 10:1 PDMS samples, we conducted bulk shear rheology measurements at values of 1%, 2%, and 3% pre-compression (uniaxial) strain. SI Figure 2(a) and (b) shows (as log-log curves) plots of G' for stiff PAH as a function of ω and γ, respectively. We focus on the limit of small strain and frequency. That is, we consider the limit $\gamma \rightarrow 0$ (for small frequency) and $\omega \rightarrow 0$ (for very small strains corresponding to the linear regime) and study the average value of G' measured for the different compression strains. Taking the zero-frequency limit provides the analogue of the static tension test data and enables comparison with the shear moduli evaluated from the Youngs's modulus and Poisson ratio from the uniaxial extension tests. The average value evaluated from the strain sweep provides data for the small but non-zero frequency and allows us to evaluate changes in the moduli due to the small (biologically relevant) frequency. Secondly, this also enables comparison with previously published data at the same frequency. The G' values measured from frequency sweep measurements were 10.7 ± 1.0 kPa, 9.80 ± 0.25 kPa, and 9.96 ± 0.2 kPa for 1%, 2%, and 3%, respectively. We find that the G' values measured from the shear strain sweeps were 10.25 ± 1.83 kPa, 8.14 ± 0.55 kPa, and 9.60 ± 3.52 kPa, for 1%, 2%, and 3% compression, respectively. These results confirm that all PAH bulk rheological responses did not significantly change with increasing compression between 1% and 3%.

The same process was done for the PDMS samples. Again, from the rheology data, we consider the limit $\gamma \rightarrow 0$ and $\omega \rightarrow 0$ and calculate the average value of G' measured for the different compression strains. PDMS

revealed increasing storage modulus as compression increased. The average *G´* values measured from frequency sweep measurements were 56.1 ± 14.7 kPa, 64.3 ± 6.8 kPa, 80.73 ± 0.99 kPa for 1%, 2%, and 3%, respectively. The average *G´* values measured from the shear strain sweeps were 67.93 ± 21.4 kPa, 84.91 ± 14.5 kPa, 93.45 ± 4.30 kPa for 1%, 2%, and 3% compression, respectively.

Table S6. Shear moduli obtained from bulk rheology with various pre-compression strains.

Sample	Compression	Shear modulus, $G'_{FrequencySweep}$ (kPa)	Shear modulus, $G'_{StrainSweep}$ (kPa)
Stiff PAH	1% compression	10.7 ± 1.0	10.25 ± 1.83
	2% compression	9.80 ± 0.25	8.14 ± 0.55
	3% compression	9.96 ± 0.2	9.60 ± 3.52
10:1 PDMS	1% compression	56.1 ± 14.7	67.93 ± 21.4
	2% compression	64.3 ± 6.8	84.91 ± 14.5
	3% compression	80.73 ± 0.99	93.45 ± 4.30

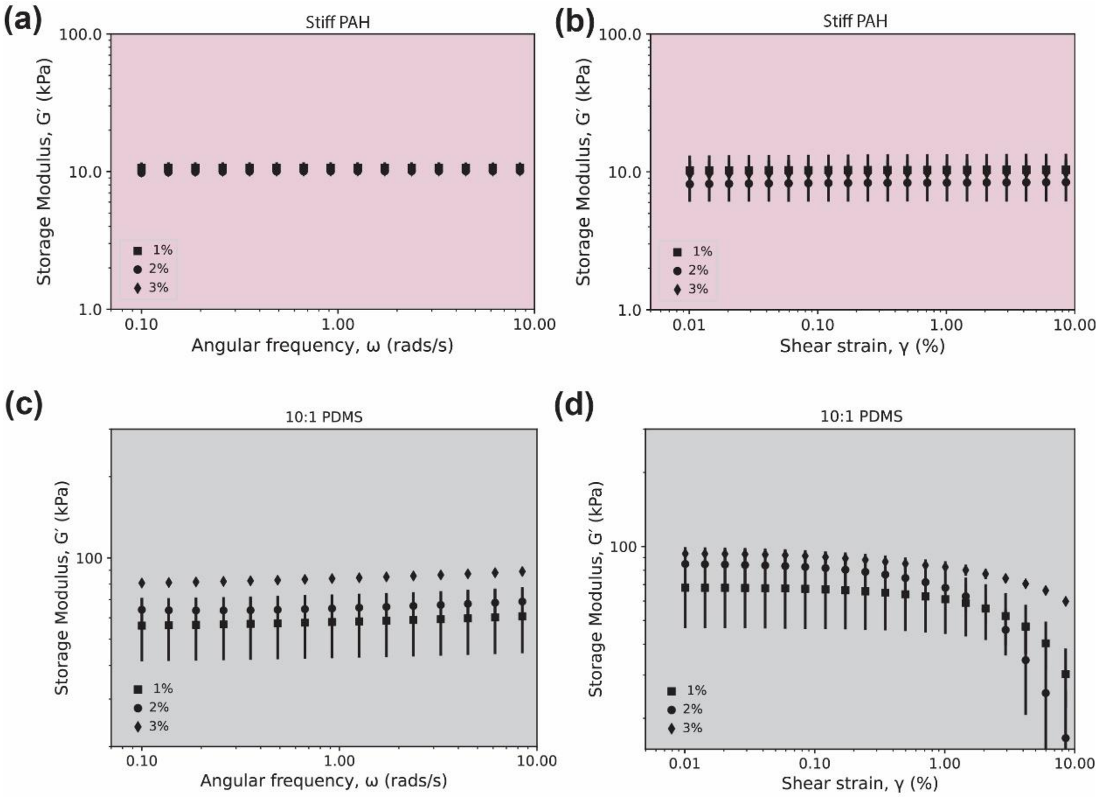

Figure S6. Effect of pre-compression strain on the bulk rheological response of stiff PAH as a function of (a) shear strain and (b) angular frequency. Effect of pre-compression strain on the bulk rheological response of 10:1 PDMS as a function of (c) shear strain and (d) angular frequency.

Table S7. Estimated mesh sizes obtained from $G_{StrainSweep}$ and $G_{FrequencySweep}$ using theories due to Flory and de Gennes. Values are obtained from the frequency sweeps, taking the zero-frequency limit.

**Sample**	**Shear modulus, $G_{FrequencySweep}$ (kPa)**	**Mesh size, ξ (nm)**
Soft PAH	2.7 ± 0.1	12
Intermediate PAH	6.6 ± 0.4	8
Stiff PAH	10.2 ± 1.1	7

SI REFERENCES

1. Cai, L.-H. *et al.* Soft Poly(dimethylsiloxane) Elastomers from Architecture-Driven Entanglement Free Design. *Adv. Mater.* **27**, 5132–5140 (2015).
2. Glover, J. D., McLaughlin, C. E., McFarland, M. K. & Pham, J. T. Extracting uncrosslinked material from low modulus sylgard 184 and the effect on mechanical properties. *J. Polym. Sci.* **58**, 343–351 (2020).
